# Factors Influencing Patients’ Intentions to Use Diabetes Management Apps Based on an Extended Unified Theory of Acceptance and Use of Technology Model: Web-Based Survey

**DOI:** 10.2196/15023

**Published:** 2019-08-13

**Authors:** Yiyu Zhang, Chaoyuan Liu, Shuoming Luo, Yuting Xie, Fang Liu, Xia Li, Zhiguang Zhou

**Affiliations:** 1 Department of Metabolism and Endocrinology The Second Xiangya Hospital Central South University Changsha China; 2 Key Laboratory of Diabetes Immunology Ministry of Education Changsha China; 3 National Clinical Research Center for Metabolic Diseases Changsha China; 4 Department of Oncology The Second Xiangya Hospital Central South University Changsha China

**Keywords:** diabetes mellitus, mobile applications, survey, structural equation modeling, China

## Abstract

**Background:**

Diabetes poses heavy social and economic burdens worldwide. Diabetes management apps show great potential for diabetes self-management. However, the adoption of diabetes management apps by diabetes patients is poor. The factors influencing patients’ intention to use these apps are unclear. Understanding the patients’ behavioral intention is necessary to support the development and promotion of diabetes app use.

**Objective:**

This study aimed to identify the determinants of patients’ intention to use diabetes management apps based on an integrated theoretical model.

**Methods:**

The hypotheses of our research model were developed based on an extended Unified Theory of Acceptance and Use of Technology (UTAUT). From April 20 to May 20, 2019, adult patients with diabetes across China, who were familiar with diabetes management apps, were surveyed using the Web-based survey tool Sojump. Structural equation modeling was used to analyze the data.

**Results:**

A total of 746 participants who met the inclusion criteria completed the survey. The fitness indices suggested that the collected data fit well with the research model. The model explained 62.6% of the variance in performance expectancy and 57.1% of the variance in behavioral intention. Performance expectancy and social influence had the strongest total effects on behavioral intention (β=0.482; *P*=.001). Performance expectancy (β=0.482; *P*=.001), social influence (β=0.223; *P*=.003), facilitating conditions (β=0.17; *P*=.006), perceived disease threat (β=0.073; *P*=.005), and perceived privacy risk (β=–0.073; *P*=.012) had direct effects on behavioral intention. Additionally, social influence, effort expectancy, and facilitating conditions had indirect effects on behavioral intention that were mediated by performance expectancy. Social influence had the highest indirect effects among the three constructs (β=0.259; *P*=.001).

**Conclusions:**

Performance expectancy and social influence are the most important determinants of the intention to use diabetes management apps. Health care technology companies should improve the usefulness of apps and carry out research to provide clinical evidence for the apps’ effectiveness, which will benefit the promotion of these apps. Facilitating conditions and perceived privacy risk also have an impact on behavioral intention. Therefore, it is necessary to improve facilitating conditions and provide solid privacy protection. Our study supports the use of UTAUT in explaining patients’ intention to use diabetes management apps. Context-related determinants should also be taken into consideration.

## Introduction

### Background

Diabetes poses heavy social and economic burdens worldwide. The estimated number of adult patients with diabetes in 2017 was 451 million worldwide, and this figure is expected to increase to 693 million by 2045 [1]. Nearly 5 million deaths in the adult population were caused by diabetes in 2017 [1]. According to a national survey in 2013, the prevalence of diabetes in China was estimated to be 10.9%, representing more than 100 million adults in China [2]. Optimal glycemic control can prevent diabetes-related complications [3]. However, in China, less than half of the patients treated for diabetes were found to have appropriate glycemic control [2]. Poor blood sugar control can lead to various life-threatening complications such as blindness, renal failure, stroke, and myocardial infarction [4]. The estimated cost of diabetes worldwide in 2015 was as high as US $1.31 trillion [5].

Diabetes self-management education and support are critical for diabetes management [6]. However, doctors in large hospitals in China are overloaded with work, and the time spent with each patient in outpatient departments is very limited and usually less than 3 min [7]. Diabetes patients receive little diabetes education in such a short time. Most patients with suboptimal glycemic control lack diabetes-related knowledge and self-care practices [8]. Moreover, due to the imbalance of medical resources in China, it is inconvenient for patients from remote rural areas to seek medical care in large hospitals [9]. Therefore, patients with diabetes in rural areas have a higher mortality [10].

Diabetes management apps enable patients to record their blood sugar, receive diabetes-related information, and communicate with health care providers and peers anytime and anywhere [11]. These apps show promising potential for diabetes self-management [12]. Several studies have shown that diabetes management apps have benefits such as glycosylated hemoglobin reduction [11,13-15], reduced feelings of loneliness [16], reduced hypoglycemic fears, and improved behavioral scores [17]. However, surveys have shown that the uptake of diabetes management apps among diabetes patients is poor. In a survey in America in 2014, the use of diabetes management apps was approximately 3.6% among Latino patients with diabetes [18]. In Australia, 8% of patients with type 2 diabetes reported using diabetes management apps [19]. Our previous Web-based survey also showed the same pattern, and only 10.8% of patients with type 2 diabetes used diabetes management apps [20]; these results were similar to those of a survey conducted in New Zealand [21].

The actual use of a technology is often determined by the intention of its use [22]. Understanding the factors that influence patients’ use intention will help manufacturers further improve the design of diabetes management apps and promote their use. However, the factors influencing patients’ intention to use diabetes management apps are unclear. Several studies have applied umbrella theoretical models to understand the determinants of use intentions for mobile health (mHealth) services [23-27] or health information technology [28]. However, a theoretical model must be identified and tested for different technologies and in different user groups, to provide a context-related understanding of technology adoption [22]. Diabetes management apps have unique functions, and patients with diabetes have unique characteristics. Therefore, it is necessary to analyze the factors influencing the use intention for diabetes management apps based on an integrated theoretical model. To our knowledge, relevant theoretical models have not been applied to the field of diabetes management apps.

### Theoretical Background

Venkatesh et al [22] integrated the following eight theories (Table 1) to form the UTAUT: technology acceptance model (TAM), theory of reasoned action, motivational model, theory of planned behavior (TPB), combined TAM and TPB, model of personal computer use, diffusion of innovations theory, and social cognitive theory. They found that the UTAUT outperformed the eight individual models [22]. The UTAUT is the most frequently used theoretical model in information technology and has been applied to a wide range of areas, such as electronic health (eHealth) services [24,29-30], electronic medical record systems [31,32] and other health-related information technologies [33,34]. According to the UTAUT, performance expectancy, effort expectancy and social influence are the core determinants of behavioral intention, and facilitating conditions and behavioral intentions are direct determinants of use behavior. Performance expectancy and effort expectancy are equivalent to relative advantage and complexity of the diffusion of innovation theory [35,36]. Venkatesh proposed the updated UTAUT2 in a consumer information technology context and found a direct association between facilitating conditions and behavioral intentions. The new model incorporates three new constructs: hedonic motivation, price value, and habit [37]. However, patients do not use diabetes management apps for the intention of enjoyment. Moreover, the Tavares and Oliveira study concerning electronic health record patient portals did not find an association between hedonic motivation and behavioral intention [38]. Diabetes management apps are offered to users for free [39] and represent a relatively new technology in China; thus, we did not incorporate the new constructs of the UTAUT2.

**Table 1:** Summary of technology acceptance theories.

Theory	Application fields	Constructs
Technology acceptance model (TAM) [23,40]	Originally designed to predict the acceptance and use of information technology, TAM has been applied to a wide range of technologies and users	Perceived Usefulness, Perceived Ease of Use, Subjective Norm
Theory of reasoned action [41]	Originating from social psychology, this model has been used widely to predict human behaviors	Attitude Toward Behavior, Subjective Norm
Theory of planned behavior (TPB) [42,43]	Extension of the Theory of Reasoned Action to deal with behaviors over which people have incomplete volitional control	Attitude Toward Behavior, Subjective Norm, Perceived Behavioral Control
Motivational model [44,45]	Widely used in psychology to explain human behavior	Extrinsic Motivation, Intrinsic Motivation
Combined TAM and TPB [46]	A hybrid model of the TPB and TAM	Attitude Toward Behavior, Subjective Norm, Perceived Behavioral Control, Perceived Usefulness
Model of personal computer use [47]	This model was adopted to predict personal computer utilization	Job Fit, Complexity, Long-Term Consequence, Affect Toward Use, Social Factor, Facilitating Conditions
Diffusion of innovations theory [48]	Grounded from sociology, this model has been applied to a wide range of innovations, such as information systems	Relative Advantage, Ease of Use, Image, Visibility, Compatibility, Results Demonstrability, Voluntariness of Use
Social cognitive theory [49]	Widely used in social behaviors, this model was also applied to information technologies	Outcome Expectations - personal, Self-efficacy, Affect, Anxiety

### Research Hypotheses

Performance expectancy, which is similar to perceived usefulness in the TAM, is defined as the degree to which use of a specific technology benefits users [37]. Several studies have shown that performance expectancy is a major determinant of the intention to use health information technologies [28,50-52]. Overall, patients tend to use eHealth tools that are beneficial for them [53]. Thus, we propose the following hypothesis:

H1: Performance expectancy positively influences the behavioral intention of patients to use diabetes management apps.

Effort expectancy is defined as the degree of the ease of use of a specific technology [37]. If patients find mHealth technology easy to use, they will have a higher intention to use it. This hypothesis has been tested in many studies [29,36-38], especially among the elderly [24,54]. Therefore, we propose the following hypothesis:

H2: Effort expectancy positively influences the behavioral intention of patients to use diabetes management apps.

The study by Alaiad [34], concerning home health care robots, found that effort expectancy is a strong determinant of performance expectancy, and the investigation of home telehealth services acceptance behavior by Cimperman al [55] also found such an association. Several other studies also revealed that performance expectancy was predicted by effort expectancy [30,56,57]. If patients find a technology easy to use, they may find it useful. Therefore, we pose the following hypothesis:

H3: Effort expectancy positively influences performance expectancy.

Facilitating conditions are defined as the consumers’ awareness of the available resources to support the use of a particular technology [37]. Although the original UTAUT model did not show a direct association between facilitating conditions and behavioral intention (showing an association between facilitating conditions and use) [22], the UTAUT2 and several other studies on the consumer environment demonstrated this relationship [24,33,37,58]. The facilitation available to each mobile app consumer can vary significantly across mobile devices and network access levels. Thus, we pose the following hypothesis:

H4: Facilitating conditions positively influence the behavioral intention of patients to use diabetes management apps.

In their study regarding telemedicine for diabetes management, Rho et al [51] showed that facilitating conditions have an indirect effect on behavioral intention, which is mediated by performance expectancy [51]. Other studies also showed that facilitating conditions can affect performance expectancy [59]. Thus, we propose the following hypothesis:

H5: Facilitating conditions positively influence performance expectancy.

Social influence is defined as the extent to which people think that others who are important to them or who can influence their behavior think that they should use a specific technology, and it is similar to the subjective norm in the TAM [22]. Studies regarding health information technologies showed that social influence affects behavioral intention [24,36]. In health care circumstances, patients’ intention to adopt a health behavior is often influenced by their doctors, peers with the same disease, and family members [60]. Thus, we propose the following hypothesis:

H6: Social influence positively influences the behavioral intention of patients to use diabetes management apps.

One study on a Web-based interactive self-management technology revealed that social influence affected behavioral intention indirectly through the mediation of perceived usefulness [61]. Since physicians are perceived as an expert authority, patients’ perceived usefulness of a health care tool is often influenced by their physician’s opinion. Thus, we propose the following hypothesis:

H7: Social influence positively influences performance expectancy.

Context can be defined as the environment in which a technology is used, and it may affect an individual’s behavioral intention [62]. The UTAUT is not derived from the environment of health information technology consumers [22,37]. According to the Health Belief Model, individuals will not take health-related actions unless they feel susceptible to or experience the severity of a disease [63]. The model has been widely employed to predict health behavioral intentions [36,63,64]. Individuals with a higher perceived health threat have greater motivation to adopt mHealth apps [62]. In this study, perceived disease threat (PDT) refers to a patient’s awareness of his/her hyperglycemia condition and concern for its potential consequences. Thus, we pose the following hypothesis:

H8: Perceived disease threat positively influences the behavioral intention of patients to use diabetes management apps.

An investigation by Ahadzadeh et al [64] found that perceived health risk and health consciousness influenced perceived usefulness of the health-related internet [64], and a study by Dou et al [65] on a hypertension management mobile app found that the perceived health threat had significant positive effects on perceived usefulness [65]. Thus, we propose the following hypothesis:

H9: Perceived disease threat positively influences performance expectancy.

Although mHealth services may improve the quality of health care and users’ quality of life, they also generate security and privacy issues [66]. The possible risks of mHealth apps include information leakage and theft. Consumers may want to use mHealth services but may not want to disclose their personal information. We define perceived privacy risk as patients’ feeling of a lack of control over their personal information after they have adopted mobile apps, and it is not consistent with a real privacy risk. Studies have shown that privacy risks negatively influence patients’ intention to use mHealth services [23,67]. Thus, we propose the following hypothesis:

H10: Perceived privacy risks negatively influence the behavioral intention of patients to use diabetes management apps.

**Figure 1:**
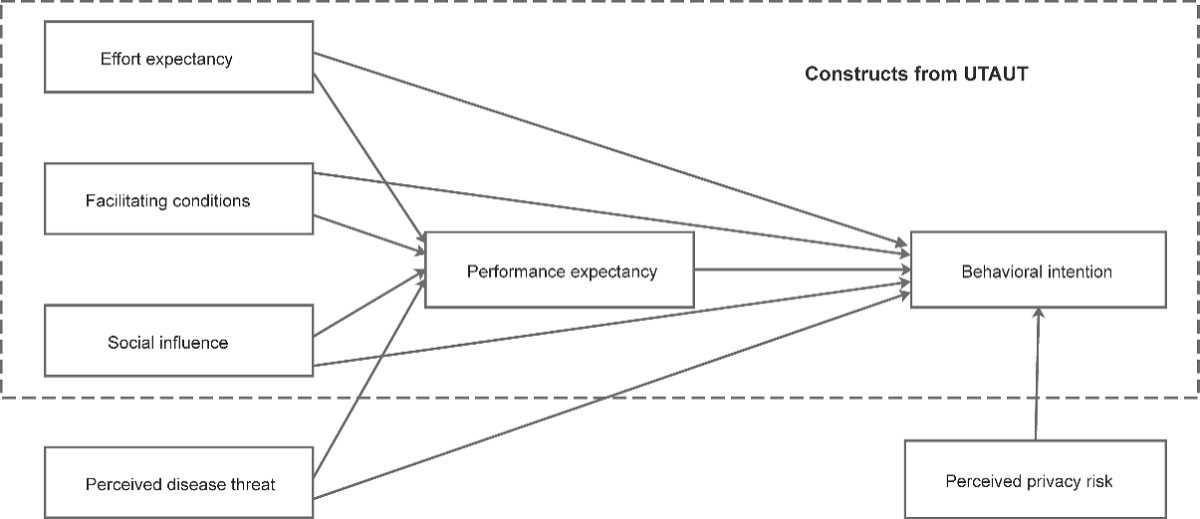
Research model. UTAUT: Unified Theory of Acceptance and Use of Technology.

The 10 research hypotheses are summarized in the research model (Figure 1).

## Methods

### Survey Instrument Design

All survey items were adopted from previous studies related to health information technology. The questionnaire items (Table 2) were translated from English to Chinese by an expert proficient in both English and Chinese, and they were discussed among an expert group selected based on their expertise and our previous explorative studies [20,68]. Items were slightly changed to adapt them to the diabetes management apps. Some items were removed or replaced to ensure face validity, content validity, and construct validity [32]. Back translation was performed from Chinese to English by another qualified translator. The items were measured with a 7-point Likert scale ranging from “strongly disagree” (1) to “strongly agree” (7).

**Table 2:** Measurement items of the constructs.

Construct		Item
**PE^a^ [29,55]**		
	PE1	Diabetes management apps help me to monitor my blood sugar.
	PE2	Diabetes management apps educate me in how to deal with my diabetes.
	PE3	Overall, diabetes management apps are useful in managing my blood sugar.
**EE^b^ [24,34,36]**		
	EE1	My interaction with diabetes management apps is clear and understandable.
	EE2	Learning how to use diabetes management apps is easy for me.
	EE3	I find diabetes management apps easy to use.
**SI^c^ [24,29,36,55]**		
	SI1	People whose opinions that I value (eg, my doctors) think I should use diabetes management apps.
	SI2	People who influence my behavior (eg, peers with diabetes) think I should use diabetes management apps.
	SI3	People who are important to me (eg, family members) think I should use diabetes management apps.
**FC^d^ [24,29,34,36]**		
	FC1	I have the resources (eg, network) necessary to use diabetes management apps.
	FC2	I have the knowledge necessary to use diabetes management apps.
	FC3	I can get help from others when I have difficulties using diabetes management apps (dropped).
**PDT^e^ [65]**		
	PDT1	I am aware that my blood sugar control is not optimal.
	PDT2	I am very concerned about my blood sugar.
	PDT3	I am very concerned about diabetes-associated complications.
**PPR^f^ [23]**		
	PPR1	I think my personal privacy information will be used for other purposes if I use diabetes management apps.
	PPR2	Because of security issues, I face the risk of personal information leakage if I use diabetes management apps.
	PPR3	I think that when I use diabetes management apps, my personal information will be abused by cyber criminals.
**BI^g^ [24,29,36]**		
	BI1	I intend to use or continue to use diabetes management apps.
	BI2	I plan to use diabetes management apps frequently.
	BI3	Overall, I have a high intention to use diabetes management apps.

^a^PE: performance expectancy.

^b^EE: effort expectancy.

^c^SI: social influence.

^d^FC: facilitating condition.

^e^PDT: perceived disease threat.

^f^PPR: perceived privacy risk.

^g^BI: behavioral intention.

We performed a pilot survey to validate the questionnaire in 10 patients with diabetes who were familiar with diabetes management apps. Context-specific adjustments were made according to the feedback from the pilot survey. On the pilot survey, patients responded that mobile apps were offered to them for free; thus, they had no opinion about the price value. Accordingly, we dropped the perceived value construct. Data on demographic characteristics such as age, sex, type of diabetes, and education level were also collected.

### Data Collection

The target population was adult patients with diabetes who were familiar with diabetes management apps. Patients under the age of 18 years and those who were unfamiliar with diabetes management apps were excluded from the survey. Data were collected using the Web-based survey tool Sojump (Changsha ran Xing InfoTech Ltd, China). From April 20 to May 20, 2019, we sent the survey link to diabetologists at hospitals collaborating in a latent autoimmune diabetes of adults study in 25 major cities in China [69]. The diabetologists shared the survey link through their WeChat contacts network. In addition to facilitating this snowball sampling, we published a survey link on three public diabetes-related WeChat accounts that had nearly 60,000 subscribed followers, and we asked patients with diabetes to complete the questionnaires. Before the survey, we introduced the purpose of the survey and explained the definition of diabetes management apps. After consent was obtained, the survey continued. The questionnaires were completed by the patients themselves. Each WeChat account and mobile IP address could complete the questionnaire only once. A set of electronic diabetes education materials was offered to participants as compensation after completing the questionnaire. The study was approved by the ethics committee of the Second Xiangya Hospital, Central South University.

### Data Analysis

The demographic characteristics of patients were analyzed by descriptive statistics. Patients’ acceptance (behavioral intention) of diabetes management apps was measured using three items (Table 2), with higher scores indicating elevated acceptance. An independent *t* test was used to evaluate the differences among acceptance scores between patients with type 1 diabetes and those with type 2 diabetes. Before evaluating the structural model, we assessed the measurement model to evaluate construct reliability, convergent validity, discriminant validity, and data fit indexes. Reliability was measured using the composite reliability and Cronbach alpha. The composite reliability and Cronbach alpha of all constructs should be greater than 0.70 [23,24]. We measured the convergent validity based on the average variance extracted (AVE) of the constructs, and the threshold was higher than 0.50 [24,65]. Discriminant validity is acceptable if the correlation coefficients between any two constructs are smaller than the square root of the corresponding AVE [62]. The model fit was generally considered acceptable when the root mean square error of approximation values was below 0.05; the ratio of χ^2^ and df was below 3; and the goodness of fit index, the adjusted goodness of fit index, the comparative fit index, the normed fit index, and the incremental fit index were above 0.90 [23,70]. The data were analyzed using SPSS [computer program] (Version 23.0. Armonk, NY: IBM Corp; 2015), and structural equation modeling analysis was performed using AMOS [computer program] (Version 23.0. Armonk, NY: IBM Corp; 2015) via a maximum likelihood estimation [32,62,71]. We performed a bootstrap analysis with 5000 bootstrap bias-corrected samples to calculate the total, direct, and indirect effects of the variables [70,72]. Values of *P*<.05 (two-tailed) were considered to indicate statistical significance.

## Results

### Sample Characteristics

**Figure 2:**
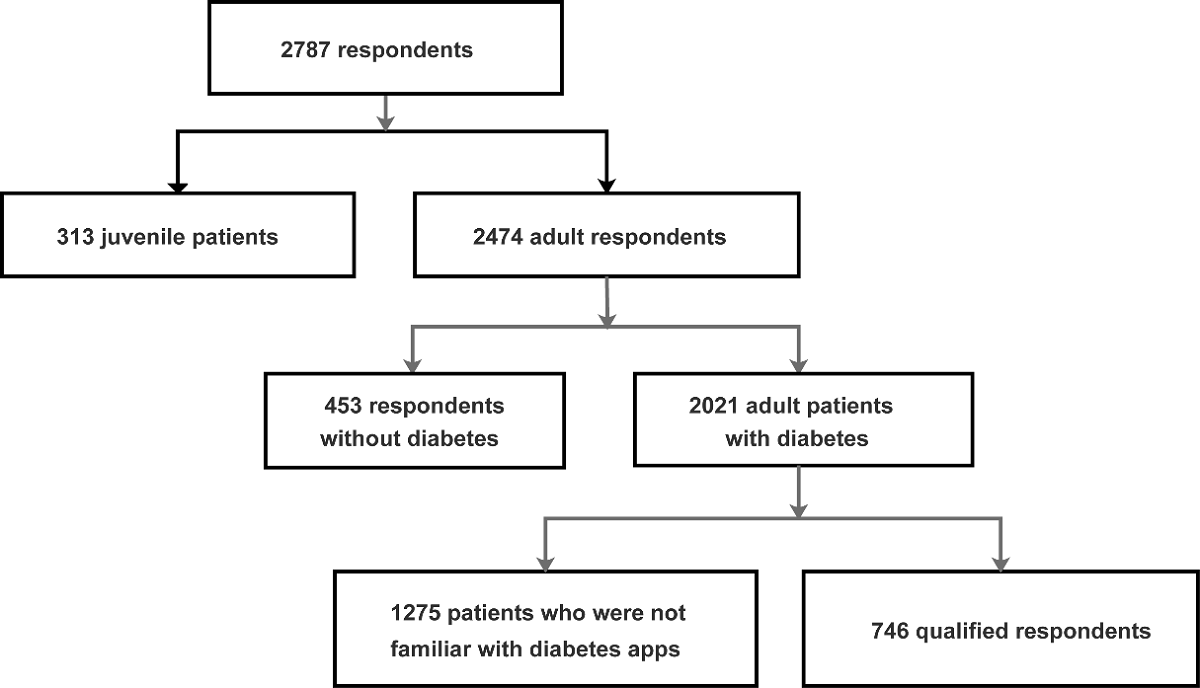
Sampling procedure and results.

Figure 2 shows the sampling procedure and results. A total of 746 participants who met the inclusion criteria completed the survey. The qualified respondent characteristics are shown in Table 3. On an average, the patients’ acceptance (behavioral intention) of diabetes management apps (min 1, max 7) was high, with a mean score of 5.65 (SD 0.99), and differences were not observed between patients with type 1 diabetes and those with type 2 diabetes (mean 5.59, SD 1.02 vs mean 5.67, SD 0.97; *P*=.097).

**Table 3:** Demographic characteristics of the qualified respondents (N=746).

Characteristics		Value, n (%)
**Gender**		
	Male	373 (50.0)
	Female	373 (50.0)
**Age (years)**		
	18-39	298 (39.9)
	40-59	349 (46.8)
	≥60	99 (13.3)
**Education level**		
	Primary school or lower	16 (2.1)
	Middle school	91 (12.2)
	High school	219 (29.4)
	University or higher	420 (56.3)
**Residence**		
	Rural	170 (22.8)
	Urban	576 (77.2)
**Diabetes type**		
	Type 1	230 (30.8)
	Type 2	455 (61.0)
	Others	33 (4.4)
	Not clearly classified	28 (3.8)
**Disease duration (years)**		
	<1	156 (20.9)
	1-4	228 (30.6)
	5-10	153 (20.5)
	>10	209 (28)

### Measurement Model Testing

One indicator (FC3) with a factor loading below 0.50 was removed [62,73]. The results of the measurement model are shown in Table 4. The composite reliability, Cronbach alpha, and AVE of each construct are greater than the recommended values, indicating good reliability and convergent validity. As shown in Table 5, the correlation coefficients between any two constructs are smaller than the square root of the corresponding AVE, indicating acceptable discriminant validity. Table 6 shows the fit indexes of the research model, which indicate that the data collected fit well with the research model.

**Table 4:** Results of the measurement model.

Constructs and items		Factor loadings	Mean score (SD)	AVE^a^	CR^b^	Cronbach alpha
**PE^c^**				0.579	0.804	0.794
	PE1	0.838	5.83 (1.05)			
	PE2	0.74	5.81 (0.87)			
	PE3	0.697	5.83 (0.93)			
**EE^d^**				0.768	0.908	0.892
	EE1	0.835	5.79 (0.99)			
	EE2	0.898	5.72 (0.99)			
	EE3	0.894	5.59 (1.01)			
**SI^e^**				0.632	0.836	0.866
	SI1	0.895	5.21 (1.13)			
	SI2	0.797	5.3 (1.13)			
	SI3	0.678	5.49 (1.10)			
**FC^f^**				0.668	0.799	0.79
	FC1	0.892	5.99 (0.87)			
	FC2	0.735	5.94 (0.88)			
**PDT^g^**				0.557	0.779	0.743
	PDT1	0.531	4.23 (1.68)			
	PDT2	0.986	5.12 (1.49)			
	PDT3	0.646	5.45 (1.43)			
**PPR^h^**				0.804	0.925	0.924
	PPR1	0.865	4.53 (1.38)			
	PPR2	0.948	4.54 (1.41)			
	PPR3	0.874	3.57 (1.35)			
**BI^i^**				0.846	0.943	0.943
	BI1	0.904	5.63 (1.03)			
	BI2	0.951	5.61 (1.07)			
	BI3	0.904	5.72 (1.05)			

^a^AVE: average variance extracted.

^b^CR: composite reliability.

^c^PE: performance expectancy.

^d^EE: effort expectancy.

^e^SI: social influence.

^f^FC: facilitating conditions.

^g^PDT: perceived disease threat.

^h^PPR: perceived privacy risk.

^i^BI: behavioral intention.

**Table 5:** Square root of average variance extracted of latent variables and correlation coefficient matrix. Italicized values represent square root of the average variance extracted; the values below them indicate the correlation coefficients.

Variable	EE^a^	SI^b^	FC^c^	PDT^d^	PPR^e^	PE^f^	BI^g^
EE	*0.876*						
SI	0.492	*0.795*					
FC	0.581	0.311	*0.817*				
PDT	–0.018	0.01	0.075	*0.746*			
PPR	–0.157	–0.238	–0.065	0.111	*0.897*		
PE	0.567	0.578	0.43	0.043	–0.211	*0.761*	
BI	0.504	0.527	0.451	0.086	–0.221	0.646	*0.92*

^a^EE: effort expectancy.

^b^SI: social influence.

^c^FC: facilitating conditions.

^d^PDT: perceived disease threat.

^e^PPR: perceived privacy risk.

^f^PE: performance expectancy.

^g^BI: behavioral intention.

**Table 6:** Fit indexes of the research model.

Fit	χ^2^/df	GFI^a^	AGFI^b^	NFI^c^	CFI^d^	RMSEA^e^	IFI^f^
Research model	2.63	0.949	0.929	0.96	0.975	0.047	0.975
Recommended value	<3	>0.9	>0.9	>0.9	>0.9	<0.05	>0.9

^a^GFI: goodness of fit index.

^b^AGFI: adjusted goodness of fit index.

^c^NFI: normed fit index.

^d^CFI: comparative fit index.

^e^RMSEA: root mean square error of approximation.

^f^IFI: incremental fit index.

### Structural Model Testing

**Figure 3:**
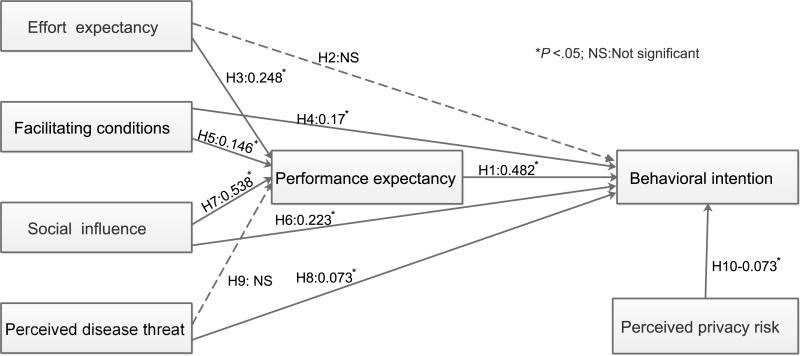
Research model explaining performance expectancy and behavioral intention (direct effects). H1: Performance expectancy positively influences the behavioral intention of patients to use diabetes management apps, H2: Effort expectancy positively influences the behavioral intention of patients to use diabetes management apps, H3: Effort expectancy positively influences performance expectancy, H4: Facilitating conditions positively influence the behavioral intention of patients to use diabetes management apps, H5: Facilitating conditions positively influence performance expectancy, H6: Social influence positively influences the behavioral intention of patients to use diabetes management apps, H7: Social influence positively influences performance expectancy, H8: Perceived disease threat positively influences the behavioral intention of patients to use diabetes management apps, H9: Perceived disease threat positively influences performance expectancy.

Figure 3 shows that 2 (H2 and H9) of the 10 research hypotheses were rejected. The nonstandardized regression weights for all other links were significant at *P*<.05. Table 7 shows the total, direct, and indirect effects (standardized regression weights) between the model variables.

Social influence, effort expectancy, and facilitating conditions explained 62.6% of the variance in performance expectancy. The effect of social influence on performance expectancy was strongest among the three variables (β=0.538, *P*=.001). Effort expectancy and facilitating conditions had moderate effects on performance expectancy (β=0.248, *P*=.003 and β=0.146, *P*=.016, respectively).

Performance expectancy had the strongest direct effect on behavioral intention (β=0.482, *P*=.001). Social influence and facilitating conditions had moderate direct effects on behavioral intention (β=0.223, *P*=.003 and β=0.17, *P*=.006, respectively). Perceived disease threat had a slight positive direct effect on behavioral intention (β=0.073, *P*=.005). Perceived privacy risk had a slight negative direct effect on behavioral intention (β=–0.073, *P*=.012). Additionally, social influence, effort expectancy, and facilitating conditions had indirect effects on behavioral intention, and these effects were mediated by performance expectancy. Social influence had the highest indirect effects among the three constructs (β=0.259, *P*=.001).

Overall, performance expectancy, social influence, disease threat, perceived privacy risk, and facilitating conditions explained 57.1% of the variance in behavioral intention. Performance expectancy and social influence had the strongest total effects on behavioral intention (β=0.482, *P*=.001). Facilitating conditions had a moderate total effect on behavioral intention (β=0.240, *P*=.001). Perceived disease threat had a slight total effect on behavioral intention (β=0.082, *P*=.002). Perceived privacy risk had a slight negative total effect on behavioral intention (β=–0.073, *P*=.012).

**Table 7:** Standardized regression weights between the model variables.

Variable		PE^a^ (R^2^=62.6%)		BI^b^ (R^2^=57.1%)	
		β	*P* value	β	*P* value
**EE^c^**					
	Direct	0.248	.003	–0.019	.85^d^
	Indirect	—^e^	—	0.119	.002
	Total	0.248	.003	0.1	.12^d^
**SI^f^**					
	Direct	0.538	.001	0.223	.003
	Indirect	—	—	0.259	.001
	Total	0.538	.001	0.482	.001
**FC^g^**					
	Direct	0.146	.02	0.17	.006
	Indirect	—	—	0.07	.01
	Total	0.146	.02	0.24	.001
**PDT^h^**					
	Direct	–0.032	.49^d^	0.073	.005
	Indirect	—	—	0.009	.46^d^
	Total	–0.032	.49^d^	0.082	.002
**PPR^i^**					
	Direct	—	—	–0.073	.01
	Indirect	—	—	—	—
	Total	—	—	–0.073	.01
**PE^j^**					
	Direct	—	—	0.482	.001
	Indirect	—	—	—	—
	Total	—	—	0.482	.001

^a^PE: performance expectancy.

^b^BI: behavioral intention.

^c^EE: effort expectancy.

^d^Not significant.

^e^Not available.

^f^SI: social influence.

^g^FC: facilitating conditions.

^h^PDT: perceived disease threat.

^i^PPR: perceived privacy risk.

^j^PE: performance expectancy.

## Discussion

### Principal Findings

Our study found that performance expectancy and social influence were the most important determinants of patients’ intention to use diabetes management apps. Several studies on mHealth services also revealed that performance expectancy was the major determinant of behavioral intention [24,65,74]. If patients with diabetes believe they can benefit from diabetes management apps, their willingness to use them will be stronger. Our previous study found that some patients were reluctant to continue using diabetes management apps because they thought the apps were useless, and the experts surveyed believed that one reason for the inefficacy of apps was their lack of comprehensiveness or functionality [20]. Physical activity, nutrition, blood glucose testing, medication, health feedback, and education are all important for diabetes management; however, few apps integrate all six diabetes management tasks [75]. Information quality is a determinant of people’s intention to seek and use health information from internet sources [76]. However, few apps provide information cited from accredited sources [77]. Blood sugar monitoring is the most frequently used feature of diabetes management apps [21]. However, patients think that merely recording their blood sugar is of little use to them [68]. Therefore, the benefits of diabetes management apps to patients are limited to a certain extent, and low performance expectancy affects patients’ willingness to use apps.

Although the direct effect of social influence on intention to use diabetes management apps was moderate, it had a significant indirect effect on behavioral intention, and this effect was mediated by performance expectancy. The effect of social influence on behavioral intention is consistent with the findings of another study on multiple sclerosis management mobile apps [74]. A survey by Hennemann et al [33] also found a prominent effect of social influence on the acceptance of Web-based aftercare. Patients’ uptake of health-related actions is susceptible to the influence of doctors, family members, and peers with the same disease. Our previous survey found that nearly half of the patients used apps because they were recommended to use them by other patients or doctors [20]. Because of the lack of clinical evidence on apps’ effectiveness, doctors do not know which apps are suitable to recommend to their patients [20]. Therefore, high-quality randomized controlled trials are needed to provide evidence-based information for doctors to recommend diabetes management apps, which will benefit the promotion of apps.

Facilitating conditions had a moderate direct effect on behavioral intention to use apps for diabetes management, and it also had a slight indirect impact on behavioral intention; this impact was mediated by performance expectancy. This result was consistent with the study of Rho et al [51] on the acceptance behavior of telemedicine for diabetes management. Despite the rapid development of smartphones in China, the use of smartphones and networks is still limited in some remote rural areas. China is vigorously advocating internet health care [78], which requires improvements to basic network facilities. App manufacturers should also provide continuous assistance services and use guidelines to support patients’ use of diabetes management apps.

Perceived disease threat had slight positive effects on patients’ intention to use diabetes management apps. The study by [65] Dou et al revealed that perceived health threat predicted patients’ intentions to use a hypertension management mobile app [65]. Several other studies concerning health information technology demonstrated the effect of disease threat on behavioral intention [29,64,71]. The awareness rate of diabetes mellitus in Chinese diabetic patients is low [2]. We should improve diabetes awareness among diabetic patients and help them correctly understand the disease. Improving patient awareness of the disease will promote patients’ intention to use diabetes management apps to manage their disease.

The negative impact of privacy risk on health information technology acceptance intention is inconsistent across studies. Our study found that perceived privacy risk had a slight negative effect on patients’ intention to use diabetes management apps. This finding is consistent with the study on mHealth services acceptance behavior [67]. A survey in America found a moderate negative effect of privacy risk on patients’ intention to use home health care robots [34]. However, a study in Bangladesh found that privacy had no effect on the adoption intention of eHealth [57]. This finding might be attributed to the different awareness levels of privacy protection in different regions. With the development of health information technology, patients are increasingly aware of privacy protection. Although our research found that perceived privacy risk has only a weak effect on the intention to use diabetes management apps, solid privacy protection measures are necessary [11].

Our study found that effort expectancy did not affect the intention to use apps for diabetes management. One study on hypertensive patients’ intention to use a hypertension management mobile app in China also did not find such an association [65]. Some studies regarding health information technology found no association between effort expectancy and behavioral intention [36,58]. However, several other studies found that effort expectancy had positive effects on behavioral intention [23,29,38], especially among the elderly [54,55]. This difference might be related to the ease of use of different technologies. However, our sample was relatively young and well educated, and some patients had been using apps for a long time, which may have biased the results.

### Limitations

First, our study used a Web-based survey. Moreover, some selection bias was unavoidable. Our surveyed patients were relatively young and highly educated; thus, a higher awareness of diabetes management apps was observed among these patients. Previous studies have demonstrated that the use of mHealth apps among younger patients and those with higher education is relatively high [20,79,80]. This bias might have influenced our results to a certain degree. For example, effort expectancy might be a determinant of the use intention among the elderly. Therefore, further offline population-based surveys are necessary, especially among the elderly. Second, our survey did not study the effect of behavioral intention on actual use. Although intention to use is a determinant of use behavior, there is usually a gap between actual use and behavioral intention [81]. However, when people have the intention to use diabetes management apps, they do not necessarily start using the apps right away. Rather, the use behavior may lag behind the intention to use it. Therefore, cross-sectional surveys may not be able to observe the impact of behavioral intention on use behavior, and further longitudinal surveys are needed to observe this impact and other factors that may affect use behavior, such as facilitating conditions. Third, our model explained only 57.1% of the variance in behavioral intention, which indicates that some other factors affecting behavioral intention may have been overlooked. Future studies could include other constructs such as compatibility of the diffusion of innovation theory [82]. Fourth, our model is for diabetes management apps, and it should be applied to other chronic disease management apps with caution. Finally, although diabetes management apps on the market are all offered for free to patients in China at present, some apps offer in-app purchases, such as diabetes education materials and telemedicine services. Therefore, the impact of perceived value on use intention needs to be further investigated.

### Conclusions

Performance expectancy and social influence are the most important determinants of patients’ intention to use diabetes management apps. Therefore, manufacturers must improve the usefulness of diabetes management apps and carry out research to provide clinical evidence for the effectiveness of these apps, which will benefit the promotion of apps. Facilitating conditions and perceived privacy risk also have an impact on behavioral intention. Therefore, it is necessary to improve facilitating conditions and provide solid privacy protection. Our study supports the use of the UTAUT in explaining patients’ intention to use diabetes management apps. In addition, context-related determinants should be considered to understand patients’ behavior intentions.

## Abbreviations

AVE: average variance extracted
BI: behavioral intention
CR: composite reliability
EE: effort expectancy
FC: facility conditions
mHealth: mobile health
PDT: perceived disease threat
PE: performance expectancy
PPR: perceived privacy risk
SI: social influence
TAM: technology acceptance model
TPB: theory of planned behavior
UTAUT: Unified Theory of Acceptance and Use of Technology
